# Assessment of the Classification of Age-Related Macular Degeneration Severity from the Northern Ireland Sensory Ageing Study Using a Measure of Dark Adaptation

**DOI:** 10.1016/j.xops.2022.100204

**Published:** 2022-07-20

**Authors:** Bethany E. Higgins, Giovanni Montesano, David P. Crabb, Timos T. Naskas, Katie W. Graham, Usha Chakravarthy, Frank Kee, David M. Wright, Ruth E. Hogg

**Affiliations:** 1 Optometry and Visual Sciences, City, University of London, London, United Kingdom; 2 National Institute for Health and Care Research, Biomedical Research Centre, Moorfields Eye Hospital, National Health Service Foundation Trust and University College London, Institute of Ophthalmology, London, United Kingdom; 3 Centre for Public Health, Queen’s University Belfast, Northern Ireland, United Kingdom

**Keywords:** AdaptDx, Age-related macular degeneration, Beckman, OCT-based grading, Rod-mediated dark adaptation, **AMD**, age-related macular degeneration, **CFP**, color fundus photography, **iAMD**, intermediate age-related macular degeneration, **RIT**, rod-intercept time, **RMDA**, rod-mediated dark adaptation, **RPE**, retinal pigment epithelium, **SDD**, subretinal drusenoid deposit, **SD-OCT**, spectral-domain OCT, **VA**, visual acuity

## Abstract

**Purpose:**

To assess the differences in rod-mediated dark adaptation (RMDA) between different grades of age-related macular degeneration (AMD) severity using an OCT-based criterion compared with those of AMD severity using the Beckman color fundus photography (CFP)-based classification and to assess the association between the presence of subretinal drusenoid deposits (SDDs) and RMDA at different grades of AMD severity using an OCT-based classification.

**Design:**

Cross-sectional study.

**Participants:**

Participants from the Northern Ireland Sensory Ageing study (Queen’s University Belfast).

**Methods:**

Complete RMDA (rod-intercept time [RIT]) data, CFP, and spectral-domain OCT images were extracted. Participants were stratified into 4 Beckman groups (omitting late-stage AMD) and 3 OCT-based groups. The presence and stage of SDDs were identified using OCT.

**Main Outcome Measures:**

Rod-intercept time data (age-corrected).

**Results:**

Data from 459 participants (median [interquartile range] age, 65 [59–71] years) were stratified by both the classifications. Subretinal drusenoid deposits were detected in 109 eyes. The median (interquartile range) RMDA for the Beckman classification (Beckman 0–3, with 3 being intermediate age-related macular degeneration [iAMD]) groups was 6.0 (4.5–8.7), 6.6 (4.7–10.5), 5.7 (4.4–7.4), and 13.2 (6–21.1) minutes, respectively. OCT classifications OCT0–OCT2 yielded different median (interquartile range) values: 5.8 (4.5–8.5), 8.4 (5.2–13.3), and 11.1 (5.3–20.1) minutes, respectively. After correcting for age, eyes in Beckman 3 (iAMD) had statistically significantly worse RMDA than eyes in the other Beckman groups (*P* ≤ 0.005 for all), with no statistically significant differences between the other Beckman groups. Similarly, after age correction, eyes in OCT2 had worse RMDA than eyes in OCT0 (*P* ≤ 0.001) and OCT1 (*P* < 0.01); however, there was no statistically significant difference between eyes in OCT0 and eyes in OCT1 (*P* = 0.195). The presence of SDDs was associated with worse RMDA in OCT2 (*P* < 0.01) but not in OCT1 (*P* = 0.285).

**Conclusions:**

Eyes with a structural definition of iAMD have delayed RMDA, regardless of whether a CFP- or OCT-based criterion is used. In this study, after correcting for age, the RMDA did not differ between groups of eyes defined to have early AMD or normal aging, regardless of the classification. The presence of SDDs has some effect on RMDA at different grades of AMD severity.

Age-related macular degeneration (AMD) is a prevalent cause of sight loss in the elderly and is characterized by progressive loss of central vision, which can worsen vision-related quality of life.^1^ Retinal imaging is used clinically to assess fundus-based structural abnormalities. The Beckman classification^2^ is a grading system that incorporates structural features detected using color fundus photography (CFP). It has been well studied using a consensus-based approach and has been extensively adopted in both clinical and research settings (including a notable ongoing clinical study^3^) because it is pragmatic and easily applied. Spectral-domain OCT (SD-OCT) provides detailed images of the macular retina and is being increasingly recognized as the imaging modality of choice for the detection of both early and late AMD features. However, longstanding AMD classification systems are based on CFP because they originated from previous epidemiologic studies,^4^^,^^5^ whereas analogous SD-OCT-based classifications were not feasible because the technology did not exist at that time.

Visual function testing has the potential to enhance the granularity of AMD staging when assessed in tandem with structural classification.^6^ Visual acuity (VA) remains standard in studies on advanced AMD; however, it has limited ability to differentiate between early and intermediate severity levels of AMD.^7^ There is accumulating evidence that another measure of visual function, namely, rod-mediated dark adaptation (RMDA), can distinguish between various stages of AMD and visually healthy peers.^6^^,^^8^^,^^9^ Abnormal (delayed) RMDA is associated with aging^10^ and is characterized by the slowed recovery of rod sensitivity to stimuli in a dark environment after light exposure that has bleached a significant proportion of visual pigment.^11^ The rate of dark adaptation is dependent on the rate of rhodopsin regeneration in photoreceptors, which in turn is dependent on the choroidal circulation, Bruch's membrane, and, crucially, functional integrity of the retinal pigment epithelium (RPE).^11^ The RPE is also thought by many (but not all) to be at the center of the AMD disease mechanism.^12^ The quantification of choriocapillaris vascular density is also an intense area of research right now because loss of choriocapillaris density is the biggest histologic effect of aging.^13^ Therefore, it is unsurprising that dark adaptation has been proposed as a functional biomarker of AMD.^14^ Delayed RMDA has been proposed as a diagnostic indicator of AMD^14^^,^^15^ that worsens with disease severity.^6^^,^^16^ Rod-mediated dark adaptation impairment has been shown to be worse in people with subretinal drusenoid deposits (SDDs)^6^^,^^9^^,^^17^ than in those without SDDs. Subretinal drusenoid deposits, which are also referred to as “reticular pseudodrusen,” are accretions of material on the inner aspect of the RPE that extend through the ellipsoid zone^18^ and are best seen using OCT rather than CFP.^19^ The presence of SDDs is considered a risk factor for atrophy and choroidal neovascularization.^20^ Histopathologic studies of eyes with SDDs have found resulting changes in retinal structure, such as shortened photoreceptor outer segments, which may explain the association between impaired RMDA and the presence of SDDs.^21^

Most studies on RMDA in people with AMD have used the presence and severity of the disease graded using CFP despite the limitation of classifications using this approach. The Beckman classification was not designed to incorporate SDDs,^18^ although CFP can be used to identify SDDs using color channel separation.^22^ The absence of an AMD classification system that includes SDDs in severity staging poses a potential issue because researchers want to further refine the status and staging of AMD. To compensate, some studies placed people with SDDs in their own independent subgroup for analysis.^6^^,^^8^^,^^9^ However, this does not illustrate the impact of the presence of SDDs on different existing severity grades.

OCT has many advantages over CFP, such as better differentiation between structural abnormalities, such as SDDs,^19^ in 3 dimensions.^23^ Our recent systematic literature review^24^ highlighted OCT-based studies that revealed new relationships between the macular anatomy of AMD (such as SDDs) and RMDA.^6^^,^^8^^,^^9^^,^^25^^,^^26^ However, the sample sizes of the SDD cohorts (n ≤ 20)^6^^,^^8^^,^^9^ were small, and few included age-adjusted control groups. This weakness of existing studies is particularly pertinent because age is a confounding variable associated with RMDA.^10^

The idea that OCT is a more accurate tool for assessing the phenotypes of AMD is not a novel viewpoint.^27^ However, the incorporation of structural abnormalities detected using SD-OCT into severity grading that cannot be readily imaged using CFP may provide a better understanding of associated RMDA impairment. To explore this idea, we took advantage of a large volume of data collected from a community-based observational study. Our primary aim was to estimate the RMDA deficits between different levels of AMD severity using an OCT-based classification and the Beckman classification based on the hypothesis that differences between AMD severity grading will be more discernible with the OCT classification. Our secondary aim was to assess the impact of incorporating the presence of SDDs into the OCT-based classification to measure the association between the presence of SDDs and RMDA metrics.

## Methods

### Participant Selection

We used prospectively collected data from a case-control study, the Northern Ireland Sensory Ageing study, which was part of the long-term, ongoing epidemiologic Northern Ireland Cohort of Longitudinal Study of Ageing study conducted at Queen’s University, Belfast. The Northern Ireland Sensory Ageing study adhered to the tenets of the Declaration of Helsinki, with ethical approval from the School of Medicine, Dentistry and Biomedical Sciences Ethics Committee, Queens University, Belfast (Ref. 14.25v4). Participants provided written informed consent before enrollment.

For the present analysis, data were included from participants aged ≥ 50 years who had complete RMDA, CFP, and SD-OCT data and had been classified using both the Beckman and SD-OCT-based grading systems (Tables S1 and S2, available at www.ophthalmologyscience.org). The full Northern Ireland Cohort of Longitudinal Study of Ageing study population had been graded according to prespecified standardized protocols. Participants with no signs of retinal disease or any early AMD features were invited to attend a second appointment for additional imaging, and a battery of psychophysical tests was performed. The details of the imaging and grading procedures are provided in the subsequent paragraphs. The exclusion criteria included presence of late-stage (geographic atrophy and/or exudative) AMD, diagnosis of any ocular disease, opaque ocular media, high refractive error ± 10 diopters, and history of squint or amblyopia. If both eyes, if applicable, were imaged and graded, only 1 eye was selected for dark adaptation assessment (eye with worse VA), and this was the study eye assessed.

### Imaging Procedures

The imaging procedures were conducted with the eyes dilated. Color fundus photography was performed using the Canon CX-1 Digital Fundus camera (Canon USA, Inc), with an environment luminance of 1.5 lux. Stereo optic disc and macular centered images were captured. The CFP images were then uploaded to a centralized reading center for secure grading and viewing using the Oculab interface (Digital Healthcare Oculab, version 3.7.98.0).

Thirty-degree volumetric SD-OCT images were taken of both eyes (61 B-scans [posterior pole] with a pattern size of 30° × 25° distance between scans of 118 μm and 11 automatic real-time tracking averaged frames), including the nondilated eye, using Spectralis SD-OCT (Heidelberg Engineering). The device uses infrared scanning laser ophthalmoscopy to track eye movements during the acquisition of images. As a result, all OCT maps can be overlaid with the infrared fundus picture. The Heidelberg Eye Explorer review software, version 1.9.17.0 (Heidelberg Engineering), segmentation system was used, and the images were visually inspected and corrected if necessary. The room luminance was previously described.

### Classification of AMD

Both eyes (if eligible) were imaged and classified into AMD stages. A single grader (T.T.N.) evaluated the SD-OCT and CFP images. A senior retina specialist (U.C.) with extensive experience in reviewing multimodal retinal images reviewed all images classified as containing RPE abnormalities, given the novelty of this phenotype, and a random selection of 10% of the remaining sample. The graders and the arbitrator were masked to all participant characteristics, including age and RMDA data. Color fundus photography-based AMD grading systems consider drusen size, location, and appearance. For more details of the Beckman clinical grading system,^2^ see Table S1. This study did not include people with late-stage (geographic atrophy and/or exudative) AMD; therefore, this Beckman stage was omitted. This study refers to Beckman stages as follows: “controls,” “early aging changes,” “early AMD,” and “intermediate AMD (iAMD)” as Beckman 0–3, respectively. Using color imaging, the presence of SDDs was assigned when a clear pattern of yellowish, interlacing ribbons or dot-like patterns were detected.

To detect and record the presence of AMD features using SD-OCT, the Heidelberg Eye Explorer software was used. On OCT images, there is currently no widely accepted method of classifying drusen according to size or volume; therefore, a simple, feature-based scheme that relies solely on the presence or absence of classical drusen, pigmentary irregularities, and SDDs was used. For more details of the OCT grading system, see Table S2 and Figure S1 (available at www.ophthalmologyscience.org). OCT classical drusen were defined as dome-shaped lesions of hyporeflectivity or medium reflectivity located between the RPE and Bruch membrane. The internal reflectivity of the largest drusen was recorded: homogenous (uniform internal reflectivity) or heterogeneous (nonhomogeneous) as described by Khanifar et al^28^; however, this information was not used in this analysis. A clear deviation of the RPE was essential to distinguish drusen from SDDs. Irregularities of the junctional components of the neurosensory retina and the inner surface of the RPE monolayer were observed, and the presence of RPE abnormalities was defined as the presence of lesions that altered the shape and structure of the RPE but could not be assigned to drusen and/or SDDs (Fig S1). Subretinal drusenoid deposits were characterized by the presence of a granular, hyperreflective material lying between the RPE and the boundary between the inside and outside sections of photoreceptors.^19^ Subretinal drusenoid deposits were graded as present, absent, or questionable (those agreed upon as “questionable” were ultimately graded as absent). A single SDD was deemed sufficient for the grading of the presence of SDDs, as per previous studies.^29^^,^^30^

After this OCT assessment, both eyes (if eligible) were allocated to 3 levels of grading: no drusen or RPE abnormalities, presence of drusen, and presence of both drusen and/or RPE abnormalities. The participants were also allocated to an additional 2 levels of grading: participants with SDDs and participants without SDDs. Subretinal drusenoid deposits were then further classified into severity stages using guidelines described by Zweifel et al^19^ (Tables S3 and S4, available at www.ophthalmologyscience.org). See Table 1 for a comparison of the 2 classifications featured in this study.

**Table 1:** Comparison of the Beckman Classification^2^ with the OCT Classification

Stage Number	Beckman Classification	OCT Classification
0	No drusen or pigmentary changes	No drusen or RPE abnormalities
1	Only drusen ≤ 63 μm, no AMD pigmentary abnormalities	Only drusen, no RPE abnormalities
2	Medium drusen > 63 μm and ≤ 125 μm, no AMD pigmentary abnormalities	Drusen and RPE abnormalities present
3	Large drusen > 125 μm and/or AMD pigmentary changes	-

AMD = age-related macular degeneration; RPE = retinal pigment epithelium.

### Standard Visual Function Measures

The best-corrected VA and contrast sensitivity were tested using a retro-illuminated ETDRS chart and the Pelli–Robson chart, respectively.

### RMDA Assessment

The eye with worse monocular VA (or right eye if both eyes had the same VA) was assigned the designated study eye. We assessed the RMDA only in the dilated study eye (with 1% tropicamide) using AdaptDx. The test was performed in a room with lights off (luminance, 0.01 lux), and the nontest eye was occluded. While the participant focused on a red fixation light, the examiner used the infrared camera to position the eye to ensure that subsequent bleaching was correctly administered. Testing commenced with the study eye bleached using exposure to a flash (duration of 0.25 milliseconds at 58 000 scotopic cd/m^2^ seconds, equivalent to a bleaching level of approximately 83% for rods); this bleached a retinal location subtending 4° centered at 5° inferiorly in the vertical meridian, consequently projected superiorly to the fovea. This was also the location of the test target. The stimuli for the threshold measurement was a diameter of 2°, a 500-nm circular target that began 15 seconds after the offset of bleaching. The participant was instructed to retain focus on the fixation light and press a hand-held button when the target first became visible in the bleached area. Log thresholds were expressed as sensitivity in decibels as a function of the time from bleaching and estimated using a modified staircase procedure (3 down/1 up). The procedure continued in intervals (30 seconds), with a break between each interval (15 seconds) until either the rod-intercept time (RIT) was met or the test protocol ended (40 minutes), whichever occurred first. The RIT is defined as the duration required for sensitivity to recover to a value of 5.0 × 10^− 3^ scotopic cd/m^2^ (3.0 log units of stimulus attenuation).^31^ In cases in which this RIT was not met, a capped value of 40 minutes was used for analysis. The device records the percentage of threshold points that indicated a fixation error. In this study, as in previous reports,^32^ if fixation errors were > 30%, the test was deemed unreliable.

### Statistical Analysis

All analyses were performed using R 3.5.2 (http://www.r-project.org/) under R Studio, version 1.1.463 (RStudio). First, we cross-tabulated the participants’ staging using the Beckman and OCT grading systems. Descriptive statistics for demographic and standard visual function measures stratified by the 2 classification methods were generated. The Kruskal–Wallis test was used to assess the differences in descriptive variables such as age. Our primary analysis focused on how the average RMDA (the RIT parameter) differed between the groups assigned using the different classification techniques. Here, we specifically used the time-to-event analysis as described in our previous work.^33^ In short, the time-to-event analysis can be conveniently used to model the time taken to recover from bleaching while accounting for predictors of interest, such as differences between groups, and correcting for covariates such as age or other attributes.^33^ Kaplan–Meier curves were plotted to visually represent the comparisons of the results from the models. Parametric time-to-event regression (using a Weibull distribution) provided in the survival package for R was implemented. We considered *P* < 0.05 as a level of statistical significance, and we corrected for multiple comparison using the Bonferroni–Holm method. This is pertinent because the CFP classification has 4 groups and 6 contrasts, whereas the OCT classification has 3 groups yielding 3 contrasts. The use of a parametric model is justified by its ability to predict the behavior of data beyond the censoring imposed by the cap in RIT recordings. Weibull models are a common choice for this type of problems, owing to their flexibility, and a strong support for any specific model does not exist for our data. Alternative distributions and their associated Akaike information criterion values are reported as a supplemental analysis, including an assessment of similarly performing distributions (Tables S6 and S7, available at www.ophthalmologyscience.org). From this analysis, it is evident that models that used a strictly positive distribution (Weibull, log-normal, log-logistic, and log-Gaussian) performed the best and, importantly, much better than the semiparametric alternative (Table S6). Therefore, any of these distributions might be an adequate, or better, description of the data. However, importantly, the specific choice of the model did not change our results (Table S7).

## Results

**Figure 1:**
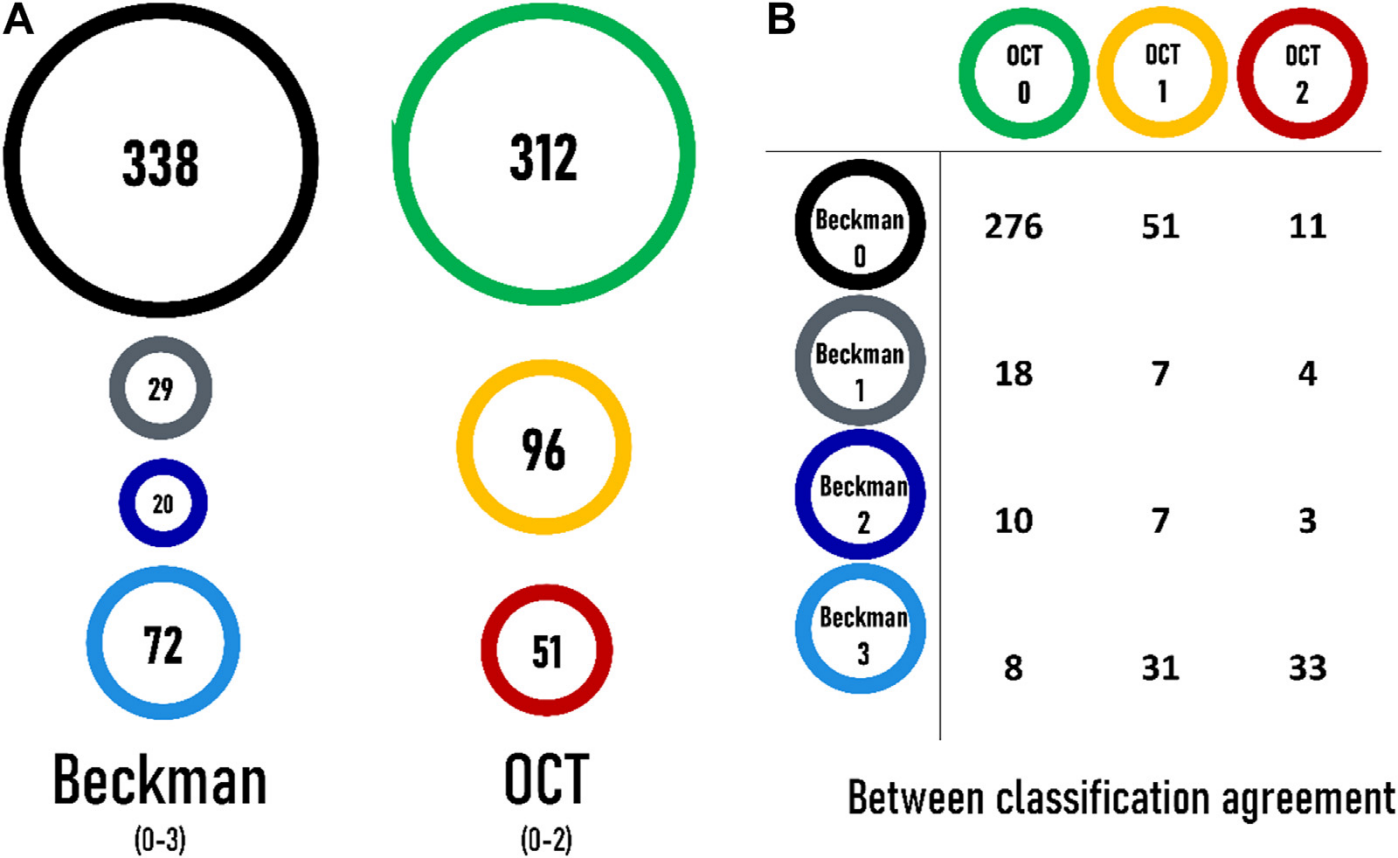
Four hundred fifty-nine participants were graded using the Beckman (0–3) and OCT classifications (0–2). **A,** Participant cohorts in each class. **B,** Agreement between the 2 classifications. For participants with the greatest discordance between the classifications (n = 8 in OCT 0 and Beckman 3 and n = 11 in OCT 2 and Beckman 0), an additional check of the grading was performed by R.E.H.. The reasons for disagreement were confirmed to be due to the different imaging technologies used, such as vitreous abnormalities detected using OCT looking like soft drusen on color, small drusen visible using OCT but not on color, and poorer-quality images on color in comparison with OCT masking subtle abnormalities on color.

Complete data were available for 459 participants (249 [54%] women), and the numbers are shown as stratified by the Beckman and OCT classifications in Figure 1. The cross-tabulation of these numbers, shown in the same figure, indicates some similarities but also some marked differences between the results of the 2 classifications. For example, 62 participants (18%; 95% confidence interval, 14%–23%) were classified as having no drusen or pigmentary changes on the Beckman scale but were shown to have some features of early AMD based on the OCT classification. Conversely, 8 participants (11%; 95% confidence interval, 5%–21%) classified as having large drusen and/or AMD pigmentary changes on the Beckman scale were observed to have no drusen or RPE abnormalities based on the OCT classification. These discrepancies show that the classifications based on CFP and OCT do not agree in all cases or, in other words, indicate that the 2 classifications provide different information on the eyes.

**Figure 3:**
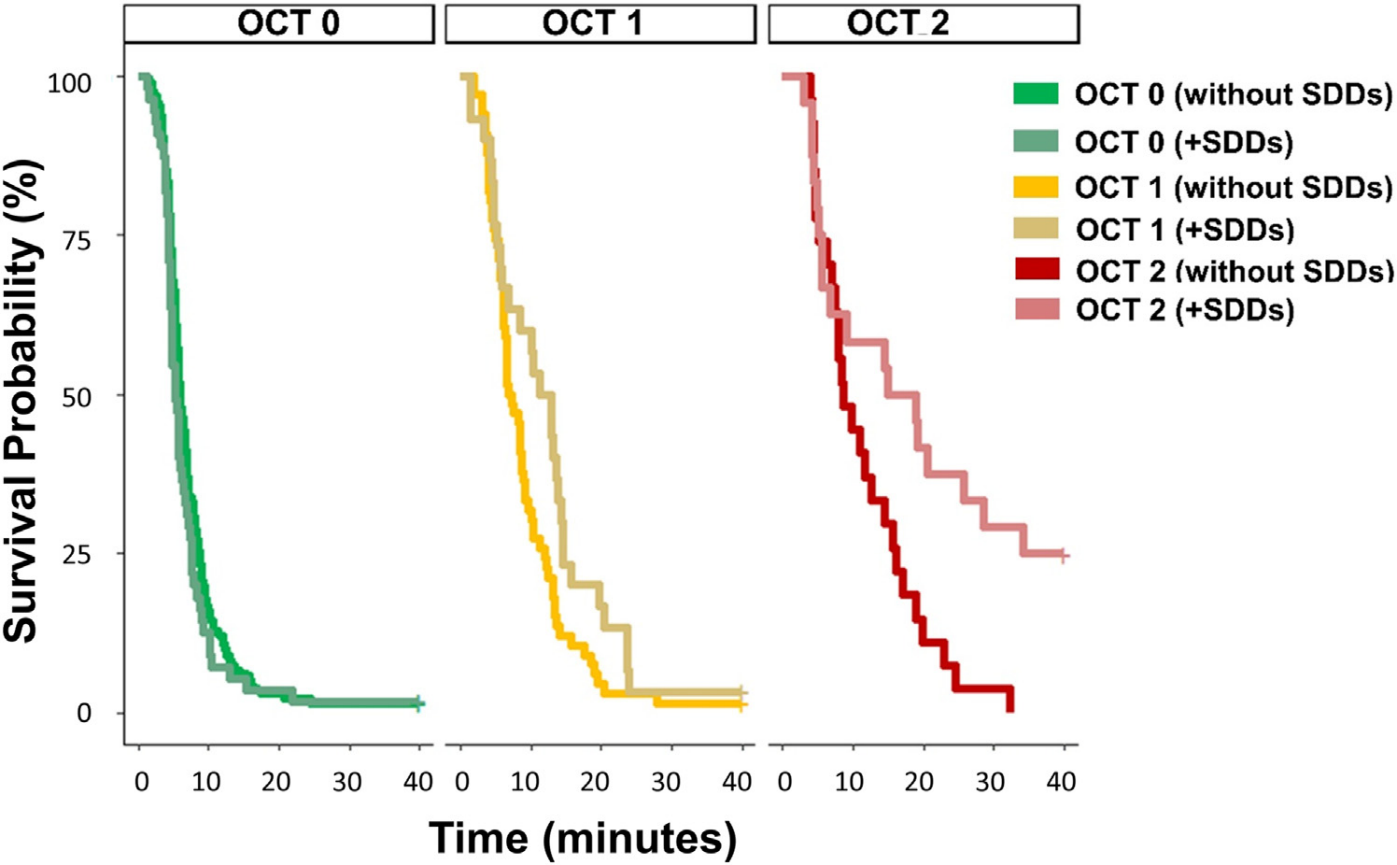
Kaplan–Meier curves illustrating the time taken for participant sensitivity to recover to a value of 5.0 × 10 ^− 3^ scotopic cd/m^2^ (3.0 log units of stimulus attenuation). This time taken is the rod-intercept time. Survival curves shown for the control and age-related macular degeneration groups stratified by the OCT classification and the presence of subretinal drusenoid deposits (SSDs).

The descriptive statistics for the demographic data and visual function variables stratified using the Beckman and OCT classifications are shown in Tables 2 and 3, respectively. The average age differences between the groups using both the classifications are noteworthy (Fig 3). There were some small but unsurprising average differences between average VA and average contrast sensitivity between some of the various groups.

**Table 2:** Summary Statistics for Demographic and Visual Function Data Stratified by the Beckman Classification

Stage Name	Stage Description	Frequency (n)	Sex (% female)	Mean (± SD)			Median (IQR)
				Age (yrs)	BCVA (Letters)	CS (LogCS)	RMDA (min)
Beckman 0	No obvious aging changes	338	54	64 (8)	85.7 (5.9)	1.6 (0.2)	**6.0 (4.5–8.7)**
Beckman 1	Normal aging changes	29	52	69 (8)	82.6 (12.7)	1.5 (0.2)	**6.6 (4.7–10.5)**
Beckman 2	Early AMD	20	75	66 (6)	84.2 (4.6)	1.5 (0.2)	**5.7 (4.4–7.4)**
Beckman 3	Intermediate AMD	72	53	71 (9)	82.0 (7.6)	1.4 (0.2)	**13.2 (6.0–21.1)**

The main visual function measure of interest was median RMDA, indicated in bold. AMD = age-related macular degeneration; BCVA = best-corrected visual acuity; CS = contrast sensitivity; IQR = interquartile range; RMDA = rod-mediated dark adaptation; SD = standard deviation.

**Table 3:** Summary Statistics for Demographic and Visual Function Data Stratified by the OCT Classification

Stage Name	Stage Description	Frequency (n)	SDDs Present (n)	Sex (% female)	Mean (± SD)			Median (IQR)
					Age (yrs)	BCVA (Letters)	CS (LogCS)	RMDA (min)
OCT 0	Controls	312	55	56%	64 (7)	86.8 (5.7)	1.6 (0.2)	**5.8 (4.5–8.5)**
OCT 1	Drusen only	96	30	53%	68 (8)	83.3 (8.9)	1.5 (0.2)	**8.4 (5.2–13.3)**
OCT 2	Drusen and/or RPE abnormalities	51	24	45%	72 (10)	82.6 (8.3)	1.4 (0.2)	**11.1 (5.3–20.1)**

The main visual function measure of interest was median RMDA, indicated in bold. BCVA = best-corrected visual acuity; CS = contrast sensitivity; IQR = interquartile range; RMDA = rod-mediated dark adaptation; RPE = retinal pigment epithelium; SD = standard deviation; SSD = subretinal drusenoid deposit.

**Figure 2:**
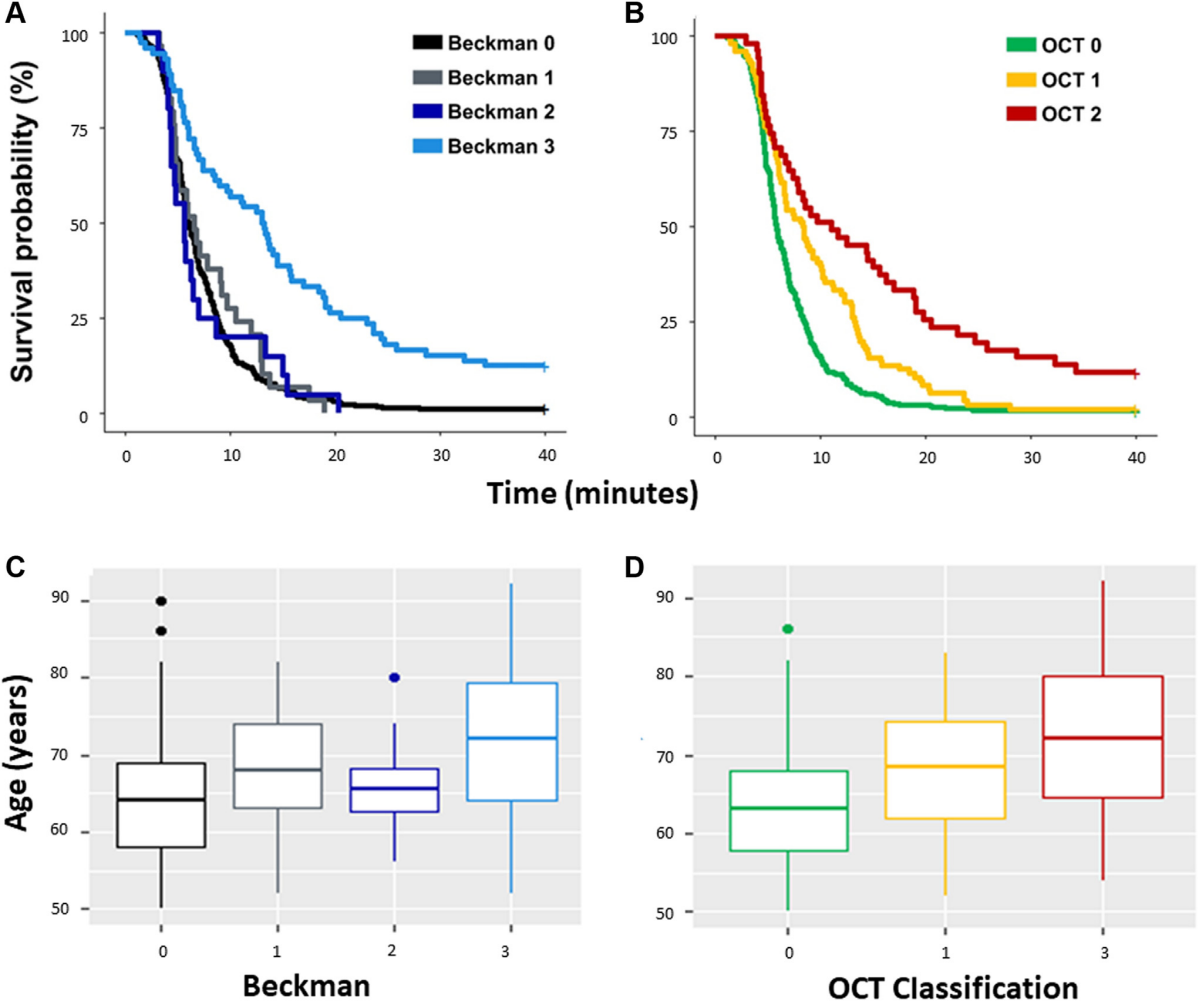
**A, B,** Kaplan–Meier curves illustrating the time taken for participant sensitivity to recover to a value of 5.0 × 10^− 3^ scotopic cd/m^2^ (3.0 log units of stimulus attenuation). This time taken is the rod-intercept time. The 2 plots show survival times for the control and age-related macular degeneration groups stratified by the Beckman (**A, C**) and OCT classifications (**B, D**). **C, D,** Age distribution for each classification. Visually, it seems like there is significant difference in the OCT classification in the rod-intercept time, but after correction for age, this is lost in earlier stages of age-related macular degeneration disease.

Our main visual function measure of interest was median (interquartile range) RMDA. In the 4 groups in the Beckman classification, i.e., Beckman 0–3, this was 6.0 (4.5–8.7), 6.6 (4.7–10.5), 5.7 (4.4–7.4), and 13.2 (6.0–21.1) minutes, respectively. The median (interquartile range) RMDA appeared different for the 3 groups in the OCT classification, being 5.8 (4.5–8.5), 8.4 (5.2–13.3), and 11.06 (5.3–20.1) minutes for OCT 0–2, respectively. These summary statistics suggest that differences in RMDA are more discernible between different grades of AMD severity when an OCT-based criterion is used compared with when the Beckman classification is used; this is illustrated by the observed separation in the time-to-event curves shown in Figure 2. Yet, these differences might be expected given that there are 4 levels of classification using the OCT-based criterion compared with just 3 in the Beckman classification.

The time-to-event analysis (uncorrected for age) indicated that only eyes with iAMD (Beckman 3) had significantly worse RMDA than eyes of each of the other groups in the Beckman classification (versus Beckman 0, 1, and 2; *P* ≤ 0.0001 for all). In contrast, no statistically significant differences were found among eyes of any of the other Beckman groups. Yet, statistically significant differences were found among all the OCT groups. Eyes in OCT 2 had worse RMDA than OCT-defined controls (OCT 0) (*P* ≤ 0.0001) and eyes in OCT 1 (*P* ≤ 0.001). Eyes in OCT 1 (presence of drusen only) had worse RMDA than the OCT-defined controls (*P* ≤ 0.001). This was in line with our observations of the “raw” median RIT data. However, the results were less clear when we subjected the data to the time-to-event analysis correcting for age. Eyes in Beckman 3 remained significantly worse than eyes in Beckman 0, 1, and 2 (*P* ≤ 0.005 for all). There also remained no statistically significant differences in the mean RMDA between any of the other pairs of groups in the Beckman classification. In contrast, although eyes in OCT 2 had a worse RMDA than eyes in OCT 0 (*P* ≤ 0.001) and OCT 1 (*P* < 0.01), the mean difference in RMDA between eyes in the OCT 0 and OCT 1 groups was not statistically significant (*P* = 0.195).

### SDD Staging and Impact on RMDA

The summary statistics in Table S2 suggest minimal differences in RMDA among people without SDDs compared with the larger differences found among people with SDDs graded based on the OCT criterion. The effect of the addition of the presence of SDDs to the time-to-event model and time-to-event curves are shown in Figure 3. The differences in the plots indicate that the presence of SDDs in each group worsens RMDA, certainly for OCT 1 and OCT 2. We formally assessed these differences by including an interaction term between the presence of SDDs and OCT grading added to the time-to-event model. The presence of SDDs significantly worsened the average RMDA in eyes in OCT 2 (*P* ≤ 0.001) and OCT 1 (*P* ≤ 0.05) but not in eyes in OCT 0 (*P* = 0.45). Once again, when we adjusted our model for age, these results were less clear. The presence of SDDs significantly worsened the average RMDA in eyes in OCT 2 (*P* ≤ 0.01) but not in eyes in OCT 1 (*P* = 0.285). On the contrary, in OCT 0, the presence of SDDs improved the RMDA (*P* < 0.05) once age adjusted.

Participants with SDDs had their SDDs graded into stages 1 to 3 (Tables S3 and S4). Because of the small number of stage 3 SDDs, only stages 1 and 2 were used for analysis (n = 99). For these participants (stage 1, n = 55; stage 2, n = 44), the median (interquartile range) RMDA was 5.3 (4.1–7.7) and 7.9 (5.1–13.9) minutes, respectively. The time-to-event model was used to assess the interaction of SDD stage with RMDA, which showed that stage 2 SDDs were associated with significant slowing of RMDA compared with stage 1 SDDs (*P* ≤ 0.01). This association between delayed RMDA and more severe stages of SDDs persisted when age was added as a covariate that was controlled for (*P* ≤ 0.05) (Fig S5, available at www.ophthalmologyscience.org).

## Discussion

We assessed how RMDA, as a reference standard functional measure, varied among the groups of eyes with different grades of AMD severity using structural measures. Differences in RMDA, as described using simple summary measures, appeared more discernible between different grades of AMD severity when an OCT-based criterion was used compared with when the Beckman classification was used. Yet, these differences might be expected given that there are 4 levels of classification using the OCT-based criterion compared with just 3 using the Beckman classification. After correcting for age, these effects were not statistically significant. To be precise, RMDA function is delayed in people with a structural definition of iAMD regardless of whether they are classified using a CFP- or OCT-based criterion; this is the main finding from our study. Rod-mediated dark adaptation does not differ in eyes defined to have less severe AMD or normal aging irrespective of whether an OCT or CFP classifier is used after correcting for age. The presence of SDDs (assessed using the OCT-based classification) has some effects on RMDA at different severity levels of AMD. The results of this study represent new knowledge about classifying people with and without AMD using structural measures. The 2 classifications provide unique information on eyes, potentially because of CFP underestimating drusen size because it essentially shows depigmentation, whereas OCT reveals the dome, seen previously.^34^ Recent insights afforded by high-resolution histologic imaging of the RPE have indicated that pigmentary changes visible in the fundus are caused by changes in RPE shape just as much, if not more so than, changes in the content or size of melanosomes and melanolipofuscin.^35^ OCT offers an alternative to CFP, which better illustrates structural changes in 3 dimensions, and incorporating it into classifiers could improve phenotyping of people with drusen and SDDs.

The idea that OCT could provide a better assessment of morphologic changes associated with AMD is not a novel one. An OCT-based classification of AMD was proposed by Lei et al,^27^ which is inclusive of high central drusen volumes, the presence of SDDs, intraretinal hyperreflective foci, and hyporeflective drusen cores. However, automated software used to assess drusen volume is machine specific (Cirrus [Carl Zeiss Meditec]) and, therefore, not widely applicable.^27^ We believe that in addition to our novel methodology that uses RMDA as our reference standard to measure visual function, this study also has important implications for both AMD classifiers and RMDA research in people with AMD.

Regardless of the structural classifier used to identify people with 10.13039/100004311AMD, our results are meaningful because they support the previously reported notion that RMDA is substantially delayed in people with iAMD compared with that in controls^6^^,^^9^^,^^25^^,^^32^ and people with early AMD.^36^ However, our results were less clear when age was considered, which is not unusual in the RMDA literature.^8^ Rod-mediated dark adaptation is affected by normal aging changes^10^ because of retinal structural changes that impact metabolic exchange between photoreceptors and the choroid.^11^ This age effect may have been underestimated in previous studies that measured RMDA in people with 10.13039/100004311AMD, as we discuss later.

Our analysis also suggests a possible structure–function relationship between the presence of SDDs visible using OCT and functional loss assessed as slowed RMDA in people with AMD. This association between the presence of SDDs and RMDA has been evidenced in the literature.^6^^,^^8^^,^^9^^,^^17^^,^^25^ For example, Flamendorf et al^6^ reported significantly worse RMDA in people who had SDDs (n = 15), with 80% reaching a test ceiling of 40 minutes. However, the patients in the small cohort with SDDs in their study were significantly older than controls; this limitation of their study is noteworthy. Although we too found that people with SDDs in the OCT 1 and OCT 2 groups had significantly worse RMDA than people without SDDs, we also went on to include age as a covariate in the time-to-event analyses. Despite the results becoming less clear, the presence of SDDs in the OCT 2 group was still associated with slower average RMDA compared with the absence of SDDs. We can infer from our data that the presence of SDDs is associated with greater rod dysfunction in people with more severe AMD, regardless of age effects. This complements previous histopathologic studies that showed that SDDs tend to be located in rod-dominated retinal locations and that the presence of SDDs has been associated with changes in photoreceptor morphology, such as shortened outer photoreceptor segments.^21^ Indeed, several studies have described how photoreceptors are shortened or bent over SDDs when multimodal imaging, including adaptive optics scanning laser ophthalmoscopy^37^ and histologic surveys, is used.^38^ However, rod dysfunction occurs where rods are sparse, such as near the rod-free fovea^39^ and where rod degeneration occurs because of aging and AMD. For example, we recently found that slowing of RMDA has been found to be worse at 5° than at 12° in people with AMD.^32^ Therefore, the presence of SDDs may not be directly related to slow RMDA but may rather serve as a marker of another process. Furthermore, we did not restrict our screening of pathology to within the RMDA testing spot, meaning that our data support previous studies that showed that the presence of SDDs is associated with delayed RMDA, regardless of whether the SDDs were in the testing location or not.^25^

We found that the presence of SDDs in the controls did not significantly impact RMDA before age adjusting the results. Similar findings were observed by Neely et al,^17^ who postulated that sparse SDD manifestations along with lack of RPE abnormalities in people without AMD was insufficient to negatively impact RMDA, suggesting that the presence of SDDs should be seen as a structural biomarker of the progression of AMD disease in controls.^9^^,^^40^^,^^41^ However, when we corrected the estimates for age, we found a surprising indication that the presence of SDDs improved RMDA (on average) in our large cohort of OCT-defined controls. We speculate that this may have been due to incomplete bleaching because of the irregular structure of the retina, which was caused by SDDs; however, there is no literature in the field to support or explain this finding. Yet, we acknowledge the discordance of this finding and believe that this would be an interesting case for future investigations performed with rigorous age matching.

Despite not being the focus of this study, we also found evidence to suggest a structure–function relationship between larger, more distinct SDDs and delayed RMDA function in people with AMD. When deposited hyperreflective materials in the interdigitation zone were sufficient enough to alter the contour of the ellipsoid zone (stage 2 SDDs),^19^ they were significantly more likely to be associated with worse RMDA compared with less “pronounced” SDDs (stage 1). This remained the case when our data were age adjusted. Because of our small sample sizes per AMD group, we could not determine whether this relationship is irrespective of AMD status. We believe this finding to be novel, however, it must be replicated in future studies to be confirmed.

The findings of this study suggest other avenues for future research. For example, the presence of SDDs and, indeed, the stage of SDD severity seem relevant while assessing functional vision, such as RMDA, and further research is critical to understand the pathophysiology of earlier stages of AMD with these structural phenomena. In addition, the presence of SDDs in controls would be the pertinent focus of future investigations to determine whether the presence of SDDs is an appropriate structural biomarker of the progression of AMD disease, given the perhaps surprising result found in this study.

We also think that our findings are relevant to the debate about designing future clinical trials looking to grade AMD. Furthermore, an OCT-based classification of AMD that takes into account the presence of SDDs would be an important tool for studies investigating the automated grading of retinal images using deep learning algorithms.^42^, ^43^, ^44^ The potential of using artificial intelligence in tandem with an OCT-based classification of AMD severity includes disease screening and therapy guidance. In fact, new imaging biomarkers have recently been discovered using deep learning algorithms in association with measures of RMDA, providing further justification for its use as an outcome measure in clinical trials.^45^

Our study has various strengths. We used a large, enriched population sample size and a large cohort of people with SDDs; this is uncommon when compared with recent RMDA research on people with AMD.^6^^,^^8^^,^^9^ Furthermore, the use of the standardized Beckman grading system for AMD allows for comparisons across relevant literature. We also utilized a time-to-event model (sometimes referred to as a survival model) to assess the magnitude of measurement differences in RMDA. This model is a statistically correct method for these data and offers advantages over alternative methods such as *t* tests and nonparametric tests, previously described.^33^ Although we were not the first to identify age as a possible confounder in RIT analyses, another strength of our methodology was that we compensated for age effects. For example, a previous study by Owsley et al^46^^,^^47^ did not correct the estimates of RIT but rather applied a correction for age and other factors to the estimated odds ratios of having an abnormal RIT based on predefined cutoffs. An approach more similar to ours was taken by Laíns et al,^25^^,^^26^ in which a multivariate model with RIT as a response variable was applied to correct for age and other factors. However, such a linear model does not account for heteroscedasticity or censoring. In fact, the authors explicitly stated that they assigned a value of 20 minutes to all observations that did not recover within the maximum allocated time. In our previous study,^33^ we showed that this introduced important distortions in the estimates of RIT. Our approach retains all the advantages of allowing for the correction of covariates while addressing fundamental properties of the specific nature of the data. The consideration of age effects in this study subsequently weakened the relationship between AMD severity and RMDA; this in itself is notable and has been demonstrated in the literature before.^8^

There are limitations to our study. For example, we only used cross-sectional data obtained from a study that was not originally designed for the purpose of testing our hypotheses. Another caveat associated with our findings surrounds the number of groups in each of the classifications that we compared. The OCT classification had 3 levels, whereas the Beckman classification had 4. Therefore, the number of eyes in each group of the Beckman classification was smaller for the statistical analysis. Furthermore, this study did not explicitly measure the interrater variability because the aim was not to definitively propose a novel grading system, and we recognize that further validation would be required. However, it is also worth noting that there is a well-analyzed lack of concordance and interrater variability in image analysis in people with AMD.^48^ In addition, there were statistically significant age differences among the AMD groups in both the classifications (Fig 3). Yet, we ameliorated this limitation by using age-corrected analyses. Additionally, although we know that size, homogeneity, and location are important while grading drusen,^22^ the incorporation of these attributes along with other OCT-based features into a grading scale has not yet been widely adopted in the literature despite efforts to create one.^27^ There also remains disagreement over the best way to stratify the features of AMD across standardized CFP-based classifications. Hence, our study featured a simple feature-based scheme using the presence or absence of classical drusen, pigmentary irregularities, and SDDs. Therefore, it is possible that a more detailed OCT-based classification that considers these additional factors may give a more distinct separation of RMDA among the groups. For example, the OCT criterion created by Lei et al^28^ incorporated intraretinal hyperreflective foci, found to be associated with progression to late-stage AMD. Intraretinal hyperreflective foci were absent from our classification, another shortcoming of this OCT criterion.

To summarize, we provided evidence to suggest that RMDA function is delayed in eyes with a structural definition of iAMD, regardless of whether they were classified using a CFP- or OCT-based criterion. In this study, RMDA did not differ between the groups of eyes defined to have early AMD or normal aging, regardless of whether the OCT or CFP classification was used after the data were age corrected. Our findings certainly add to the debate about how we stratify the severity of AMD disease. For example, the presence of SDDs was evidenced to have some effect on RMDA at different levels of AMD severity using the OCT classification.
