# Supplementary material for: Assessment of the Classification of Age-Related Macular Degeneration Severity from the Northern Ireland Sensory Ageing Study Using a Measure of Dark Adaptation
Source: Ophthalmol Sci. 2022 Jul 20;2(4):100204. doi: 10.1016/j.xops.2022.100204 (PMC9754971; doi:10.1016/j.xops.2022.100204)
Supplement: Table S1 [file mmc1.pdf]

Table 1 The Beckman Classification of AMD severity, adapted from Ferris et al (2013)

**Beckman Stage Number**	**Beckman Stage Name**	**Definition (areas of lesions within 2 disc diameters from the foveal centre)**
0	No obvious ageing changes	No Drusen – No pigmentary changes*
1	Normal aging changes	Only Drusen ≤63µm – No AMD pigmentary abnormalities*
2	Early AMD	Medium drusen >63µm and ≤125µm – No AMD pigmentary abnormalities*
3	Intermediate AMD	Large Drusen >125µm - any other AMD pigmentary changes*

*AMD pigmentary changes = any definite hyper- or hypopigmentation with medium or large drusen not associated with any known disease entities
