# Supplementary material for: Assessment of the Classification of Age-Related Macular Degeneration Severity from the Northern Ireland Sensory Ageing Study Using a Measure of Dark Adaptation
Source: Ophthalmol Sci. 2022 Jul 20;2(4):100204. doi: 10.1016/j.xops.2022.100204 (PMC9754971; doi:10.1016/j.xops.2022.100204)
Supplement: Table S2 [file mmc2.pdf]

Table 2. The OCT Classification of AMD severity

Stage Number	Stage Description	SDDs	Frequency	Mean Age in years (SD±)	Median RMDA (IQR)
0	Controls	No	257	63 (7)	6.0 (4.6, 8.7)
		Yes	55	66 (9)	5.3 (4.4, 7.6)
1	Drusen only	No	66	66 (8)	7.1 (5.1, 11.8)
		Yes	30	73 (7)	12.1 (5.3, 14.6)
2	Drusen and/or RPE abnormalities	No	27	69 (10)	8.6 (5.7, 15.9)
		Yes	24	75 (8)	17.0 (5.4, 35.7)

Subretinal drusenoid deposits (SDDs); Interquartile Range (IQR), Standard Deviation (SD)
