# Supplementary material for: Assessment of the Classification of Age-Related Macular Degeneration Severity from the Northern Ireland Sensory Ageing Study Using a Measure of Dark Adaptation
Source: Ophthalmol Sci. 2022 Jul 20;2(4):100204. doi: 10.1016/j.xops.2022.100204 (PMC9754971; doi:10.1016/j.xops.2022.100204)
Supplement: Table S3 [file mmc3.pdf]

Table 3. Subretinal drusenoid deposit classification by Zweifel et al. (2010).

Stage Number	Description
0	Diffuse deposition of granular hyperreflective material in the interdigitation zone
1	Mounds of accumulated material sufficient to alter the contour of the ellipsoid zone
2	Material with conical appearance breaking through the ellipsoid zone
