# Supplementary material for: Assessment of the Classification of Age-Related Macular Degeneration Severity from the Northern Ireland Sensory Ageing Study Using a Measure of Dark Adaptation
Source: Ophthalmol Sci. 2022 Jul 20;2(4):100204. doi: 10.1016/j.xops.2022.100204 (PMC9754971; doi:10.1016/j.xops.2022.100204)
Supplement: Table S4 [file mmc4.pdf]

**Table 4**. SDD presence stratified using OCT classification and Zweifel et al (2010) SDD staging

| OCT Group | Participants with SDDs | SDD Stage | | |
|---|---|---|---|---|
| | | **Stage 1** | **Stage 2** | **Stage 3** |
| 0 | 55 | 34 | 20 | 1 |
| 1 | 30 | 14 | 13 | 3 |
| 2 | 24 | 7 | 11 | 6 |

Optical coherence tomography (OCT); Subretinal drusenoid deposits (SDDs)
*Due to small number of Stage 3 SDDs, these were omitted from further analysis
