# Supplementary material for: Assessment of the Classification of Age-Related Macular Degeneration Severity from the Northern Ireland Sensory Ageing Study Using a Measure of Dark Adaptation
Source: Ophthalmol Sci. 2022 Jul 20;2(4):100204. doi: 10.1016/j.xops.2022.100204 (PMC9754971; doi:10.1016/j.xops.2022.100204)
Supplement: Table S6 [file mmc5.pdf]

Table 6. AIC values for both age-corrected survival models for both classifications using all available distributions

Classification	Distribution	Type of distribution	AIC
**Beckman**	**Weibull**	**Parametric**	**2607**
**OCT**	**Weibull**	**Parametric**	**2625**
Beckman	Cox	Semi-parametric	4549
OCT	Cox	Semi-parametric	4555
Beckman	Exponential	Parametric	2787
OCT	Exponential	Parametric	2792
Beckman	Gaussian	Parametric	2974
OCT	Gaussian	Parametric	2994
Beckman	Logistic	Parametric	2825
OCT	Logistic	Parametric	2839
**Beckman**	**Lognormal**	**Parametric**	**2509**
**OCT**	**Lognormal**	**Parametric**	**2516**
**Beckman**	**Loglogistic**	**Parametric**	**2473**
**OCT**	**Loglogistic**	**Parametric**	**2484**
Beckman	Rayleigh	Parametric	2640
OCT	Rayleigh	Parametric	2667
**Beckman**	**Loggaussian**	**Parametric**	**2509**
**OCT**	**Loggaussian**	**Parametric**	**2516**
Beckman	t	Parametric	2722
OCT	t	Parametric	2733
Beckman	Extreme	Parametric	3313
OCT	Extreme	Parametric	3344

Analysis was repeated using different parametric and semi-parametric distributions, with age corrected for. Lognormal, loglogistic and loggaussian distributions had lower Akaike information criterion (AIC) values, so pairwise comparisons between the variables in the survival models were computed to assess the best model to use.
