# Supplementary material for: Assessment of the Classification of Age-Related Macular Degeneration Severity from the Northern Ireland Sensory Ageing Study Using a Measure of Dark Adaptation
Source: Ophthalmol Sci. 2022 Jul 20;2(4):100204. doi: 10.1016/j.xops.2022.100204 (PMC9754971; doi:10.1016/j.xops.2022.100204)
Supplement: Table S7 [file mmc6.pdf]

Table 7. Pairwise comparisons between classification levels within the age-corrected survival model using different distributions

Pairwise comparisons between variables (age-corrected)	Distribution	*P* values (Bonferroni Holm correction)
Beckman 0-1	Weibull	1
Beckman 0-2	Weibull	1
Beckman 0-3	Weibull	**<.0001**
Beckman 1-2	Weibull	1
Beckman 1-3	Weibull	**<.001**
Beckman 2-3	Weibull	**<.01**
Beckman 0-1	Lognormal	1
Beckman 0-2	Lognormal	1
Beckman 0-3	Lognormal	**<.0001**
Beckman 1-2	Lognormal	1
Beckman 1-3	Lognormal	**<.01**
Beckman 2-3	Lognormal	**<.05**
Beckman 0-1	Loglogistic	1
Beckman 0-2	Loglogistic	.746
Beckman 0-3	Loglogistic	**<.0001**
Beckman 1-2	Loglogistic	1
Beckman 1-3	Loglogistic	**<.001**
Beckman 2-3	Loglogistic	**<.001**
Beckman 0-1	Loggaussian	1
Beckman 0-2	Loggaussian	1
Beckman 0-3	Loggaussian	**<.0001**
Beckman 1-2	Loggaussian	1
Beckman 1-3	Loggaussian	**<.01**
Beckman 2-3	Loggaussian	**<.01**
OCT 0-1	Weibull	.20
OCT 0-2	Weibull	**<.001**
OCT 1-2	Weibull	**<.01**
OCT 0-1	Lognormal	.082
OCT 0-2	Lognormal	**<.001**
OCT 1-2	Lognormal	**<.05**
OCT 0-1	Loglogistic	.062
OCT 0-2	Loglogistic	**<.001**
OCT 1-2	Loglogistic	.062
OCT 0-1	Loggaussian	.082
OCT 0-2	Loggaussian	**<.001**
OCT 1-2	Loggaussian	**<.05**

Despite having a higher AIC, there is no material difference between the p-values therefore it is appropriate for our analysis to use the Weibull distribution
