# Supplementary material for: Assessment of the Classification of Age-Related Macular Degeneration Severity from the Northern Ireland Sensory Ageing Study Using a Measure of Dark Adaptation
Source: Ophthalmol Sci. 2022 Jul 20;2(4):100204. doi: 10.1016/j.xops.2022.100204 (PMC9754971; doi:10.1016/j.xops.2022.100204)
Supplement: Figure S1 [file mmc7.pdf]

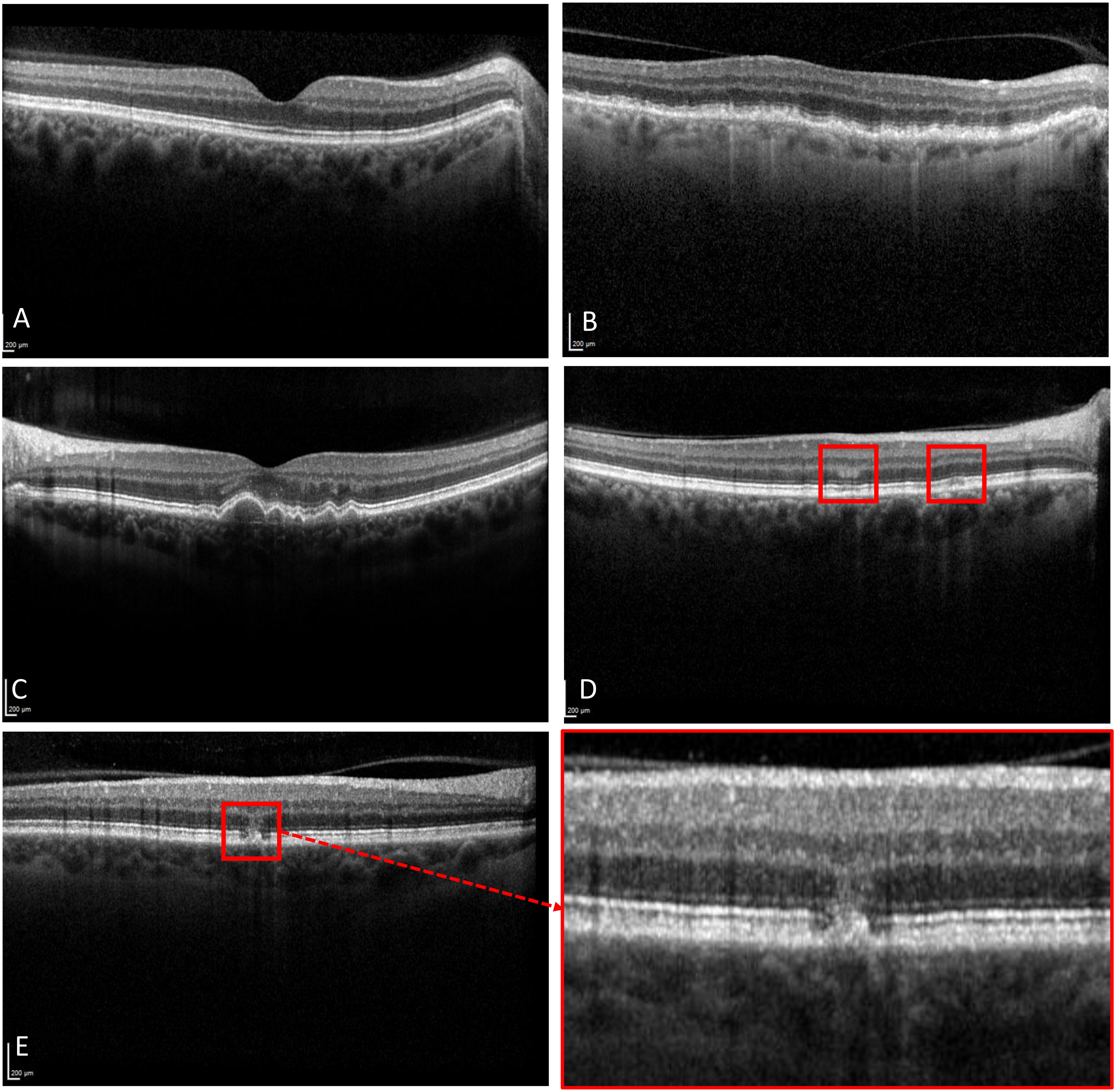

Figure 1. Representative OCT scans of study population. A: Scan from a healthy participant, all layers are discernible. B: SDD case. C: Intermediate AMD case with large drusen. D: RPE abnormality case. E: RPE abnormality case with magnified insert of abnormality. RPE abnormality was defined as the presence of lesions that altered the shape-structure of the RPE but could not be assigned to drusen and/or SDD. No cases of CNV or GA are given as these were excluded.
