# Supplementary material for: Assessment of the Classification of Age-Related Macular Degeneration Severity from the Northern Ireland Sensory Ageing Study Using a Measure of Dark Adaptation
Source: Ophthalmol Sci. 2022 Jul 20;2(4):100204. doi: 10.1016/j.xops.2022.100204 (PMC9754971; doi:10.1016/j.xops.2022.100204)
Supplement: Figure S5 [file mmc8.pdf]

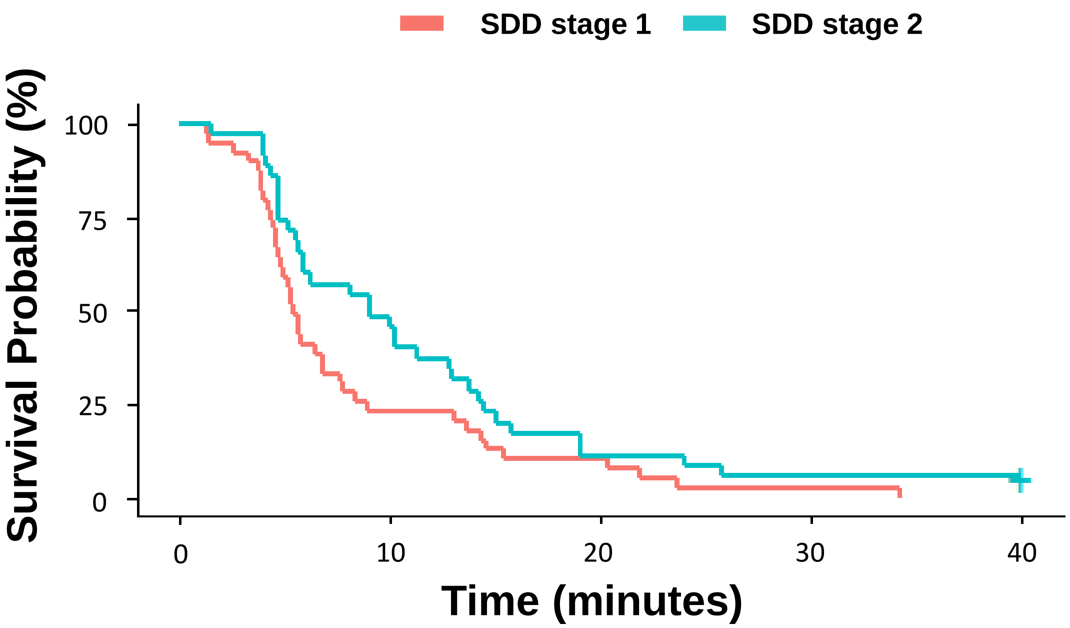

Figure 5. Kaplan-Meier curves illustrating the time taken for participant sensitivity to recover to a value of 5.0 x 10^{-3} scotopic cd/m^2 (3.0 log units of stimulus attenuation). This time taken is the RIT. Survival curves shown for people with stage 1 SDDS and people with stage 2 SDDS
